# Periconceptional Folate Deficiency and Implications in Neural Tube Defects

**DOI:** 10.1155/2012/295083

**Published:** 2012-08-05

**Authors:** J. Safi, L. Joyeux, G. E. Chalouhi

**Affiliations:** ^1^Department of Obstetrics and Gynecology, St Joseph University, Hôtel-Dieu de France, 1100 Beirut, Lebanon; ^2^Department of Pediatric Surgery, Children's Hospital of Dijon, University Medical Center, 21000 Dijon, France; ^3^Department of Obstetrics and Fetal Medicine, Hôpital Necker-Enfants-Malades, Paris Descartes University, Assistance Publique-Hôpitaux de Paris, 75015 Paris, France

## Abstract

Nutritional deficiencies are preventable etiological and epigenetic factors causing congenital abnormalities, first cause of infant mortality. Folate deficiency has a well-established teratogenic effect, leading to an increasing risk of neural tube defects. This paper highlights the most recent medical literature about folate deficiency, be it maternal or paternal. It then focuses on associated deficiencies as nutritional deficiencies are multiple and interrelated. Observational and interventional studies have all been consistent with a 50–70% protective effect of adequate women consumption of folates on neural tube defects. Since strategies to modify women's dietary habits and vitamin use have achieved little progress, scientific as well as political effort is mandatory in order to implement global preventive public health strategies aimed at improving the alimentation of women in reproductive age, especially folic acid supplementation. Even with the recent breakthrough of fetal surgery for myelomeningocele, the emphasis should still be on prevention as the best practice rather than treatment of neural tube defects.

## 1. Introduction

 Congenital abnormalities (CAs) concern all diseases of organs or body parts developed in utero. They can be either isolated localized in one organ or multiple affecting at least two organs grouped into a syndrome, a sequence, or an association. Their prevalence is about 14% of all human fetuses considering all types of abnormalities, that is, major (3%) and minor (11%), or lethal, severe, and benign [1]. Among major CAs, congenital heart diseases account for 25%, limb defects for 20%, and nervous system abnormalities for 10% [2]. Moreover, CAs represent the first cause of infant mortalities, with an increasing proportion (more than 25%) in both developed and developing countries [1, 3]. In 2002 in the USA, CAs caused 21% of infant deaths [4, 5]. In the world, more than 10% of infant mortalities secondary to CA are caused by nervous system abnormalities [1].

 Congenital abnormalities can develop at any time after the first month of pregnancy. From conception to birth, the human egg, then the embryo, and the fetus have to adapt, at a molecular and transcriptional level, to various changes in their cellular environment. At conception, this environment depends on the micronutritional status of maternal and paternal germ cells and after conception on maternal nutritional status, metabolism, and lifestyle. Maternal diet is the source of all the essential elements that will serve as basic components, transcriptional factors, growth factors, and messengers for embryological and fetal cells signaling and development.

 Prevention of CAs is defined by individual and public health strategies that can reduce their prevalence. These active strategies include nutritional interventions, prevention of maternal infections and diseases, periconceptional care of sick mothers (epileptic or diabetic), control of professional and environmental exposure to teratogens, and special attention to pregnancies exposed to major health determinants such as obesity, tobacco, alcohol, and drugs [6].

 One of the major breakthroughs in CA prevention has been the evidence that periconceptional folate supplementation can reduce the risk of neural tube defects (NTDs) [7–10] and other congenital abnormalities like cardiovascular malformations (CVMs), cleft lip and palate [11], urogenital abnormalities, and limb reductions [12]. It is essential to point out here that NTDs preventable through folate supplementation are isolated NTDs, and exclude other associated NTDs—grouped in syndrome, sequence, or association of CAs—which do not fall within the scope of this paper.

## 2. Nutritional Deficiencies and Teratogenicity

 Congenital heart and central nervous system abnormalities encompass approximately 50% (resp., 40 and 10%) of the worldwide infantile deaths attributable to congenital abnormalities [13, 14]. Major congenital anomalies are also a source of high morbidity, distress, and severe physical, psychological, and social handicaps [13].

**Figure 1:**
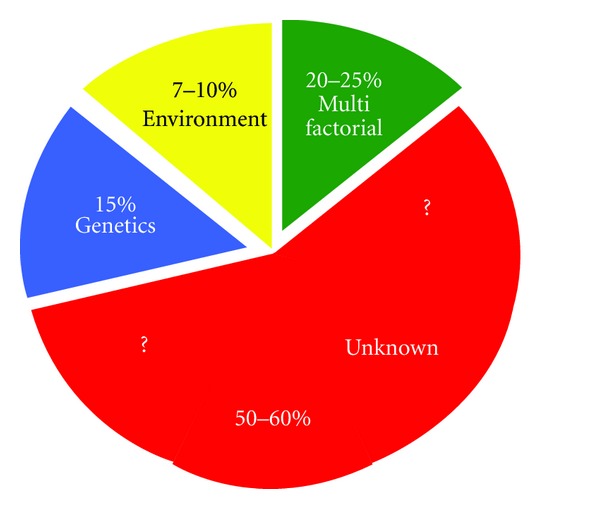
Causes of human congenital abnormalities (adapted from [65]).

 Teratology, the science of the precise etiologies of CA, defines these causes as unknown in 50–60% of cases. The other etiologies are epigenetic and multifactorial in 20–25% of cases, chromosomic or genetic with a single gene mutation in almost 15% of cases, and epigenetic, acquired, and monofactorial under the influence of environmental factors (such as maternal sickness, infections, medications, ionizing radiations, and alcohol) in about 10% of cases [14] (Figure 1).

 Clinical studies [3] have revealed that a specific teratogen can induce various malformations, or none, depending on the timing of exposure of the developing embryo. Thereby, each organ or system displays a critical, yet brief, window, considered as a phase of susceptibility to environmental teratogens. It is commonly known that the earlier the exposure, the more severe the abnormalities, which can even lead to death of the embryo during the first month postconceptionally. Most deleterious teratogens produce nonspecific congenital abnormalities such as general dysmorphic features, intrauterine demise, or intrauterine growth restriction, as well as specific CA, which can characterize a particular agent. Nevertheless, a specific CA can result from various environmental agents. For example, spina bifida occurrence is increased with three principal maternal risk factors and still exhibits the same clinical aspect: maternal valproic acid intake, insulin-dependent diabetes, and folate deficiency.

 Improved comprehension of etiopathogenesis has led to the emerging evidence that equilibrating and optimizing maternal dietary intake can reduce the incidence of CA. Influence of nutrition on fetal development has been repeatedly proven, at the molecular as well as the clinical level. Very recently, pilot data from a randomized double blind controlled trial showed that periconceptional maternal micronutrient supplementation affected fetal genome methylation patterns in DNA samples drawn from cord blood [15]. Any nutritional imbalance can alter genotype expression and induce abnormal phenotype. This is the fundamental epigenetic gene/nutrients link.

## 3. Characteristics of Folate Deficiency

 Folate, or vitamin B9, is most abundantly found in dark green leafy vegetables, but also in orange juice, legumes (e.g., black beans and kidney beans), nuts, asparagus, and strawberries. With the exception of liver, meat is not a good source of folate [16]. Folic acid is the synthetic form of folate and is usually more bioavailable than natural food folate. Due to its lower bioavailability from natural foods, many countries have adopted mandatory folic acid food fortification programs.

 Folates are essential for the synthesis of thymidylate and purines, precursors required for *de novo* DNA synthesis and hence, cell division [17]. This feature is of particular importance in a rapidly dividing and developing embryo. Folate coenzymes are also implicated in amino acid metabolism (homocystein) and methylation. In order to be stored intracellularly, folate ought to be metabolized into tetrahydrofolate (THF) by methionine synthase, a B12-dependent enzyme. In humans, the association of CA and folate deficiency began to be acknowledged in the 1950s, when Methotrexate was widely used for abortions. Moreover, Methotrexate and Aminopterin, both folic acid antagonists, were being used for the treatment of psoriasis and certain cancers in pregnant women, which resulted in CA, thus starting to reveal an association. Additional research into neural tube defects and their etiologies was further facilitated by the advances in genetics and nonmendelian complex diseases, paving the way for the study of metabolism and transport of folate-homocysteine as potential risk factors for spina bifida. Since then, folic acid supplementation has remained one of the few interventions, if not the only, that can prevent major CA in the human fetus.

### 3.1. Maternal Folate Deficiency

 During pregnancy, folate requirements increase to accommodate embryonic and fetal development and maternal tissue growth. While folate is actively transported to the fetus as demonstrated by higher cord blood folate concentrations relative to maternal blood [18], maternal serum and RBC concentrations of folate decline for several reasons [19–24]: increased demand, dilution secondary to increased intravascular volume, increased folate catabolism and clearance, decreased absorption, and inadequate intake [19, 22]. Folate deficiency is known to lead to maternal megaloblastic anemia, which may be fatal if left untreated [25].

#### 3.1.1. Prevention of NTDs

 Addressing folate deficiency as it relates to NTDs occurrence or recurrence has been the subject of considerable study. The evidence in public health that daily folic acid supplementation (alone or in combination with other vitamins and minerals) has a significant protective effect in preventing NTDs, that is, anencephaly and spina bifida as well as cardiovascular malformations is now overwhelming [26]. There is also a significant reduction in risk of recurrence, while prevention of other birth defects (cleft palate cleft lip) and or miscarriages was not proven to be statistically significant. The controversy about the potential role played by folic acid supplementation in the rising colon cancer rates should no longer be defended, as the majority of the evidence available is reassuring [25].

**Figure 2:**
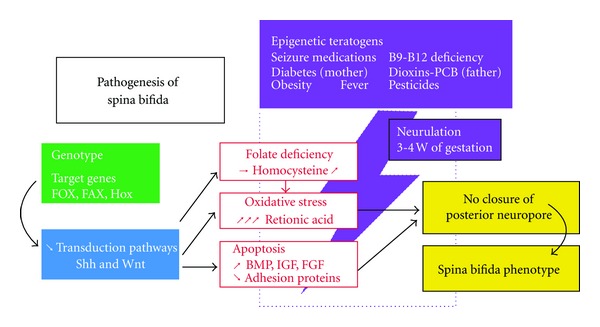
Combined etiopathogenesis of spina bifida (Shh: sonic hedgehog, Wnt: Wnt ligand in Wnt/*β* catenin signaling pathway, BMP: bone morphogenic protein, IGF: insulin-like growth hormone, and W: weeks) [66].

 The current identified maternal risk factors for NTDs include four established factors: personal or familial past history of NTD (relative risk RR of 30), maternal diabetes (RR, 2–10), certain antiseizure medications (carbamazepine and valproic acid, with RR of 10 to 20), and maternal folate deficiency (RR, 2–8) [27] (Table 1 and Figure 2). Maternal factors such as obesity, hyperthermia, race, ethnicity, smoking, alcohol abuse, malabsorption, intestinal disease, and liver or renal failure, can also contribute to genesis of NTDs either directly or indirectly by folate deficiency [25, 28]. Low folate intake, in addition to inadequate absorption of food folate and further loss through cooking practices, leaves the majority of women of reproductive age deficient in folates. As closure of the developing neural tube occurs by the 28th postconceptional day, that is, the 42nd gestational day, before the majority of women are aware of their pregnancy, this precludes the efficacy of folic acid given after the diagnosis of pregnancy.

 Based on current evidence, it is recommended that all women of childbearing age receive 0.4 mg (400 *μ*g) of folic acid daily periconceptionally (1 month before and 2 months after). Women at high risk for NTDs, that is, women with previous NTD-affected pregnancy, obesity (BMI > 30), diabetes, and epilepsy, should receive 4 to 5 mg of folic acid daily preconceptionally, starting at least one month before conception and continuing throughout the first trimester of pregnancy [25]. The recommended dose of 4 mg/d was chosen for a Medical Research Council trial that resulted in a 72% reduction in NTD recurrence [16, 29]. The adequate blood folate concentration and minimum supplemental dose shown to be effective for the prevention of NTDs are not precisely known. The only Randomized Controlled Trial (RCT) showing reduction in *occurrence* of NTDs used an 800 *μ*g/d dose, whereas other intervention trials studying occurrence or case control studies of *recurrence* used a 400 *μ*g/d dose. All of the studies demonstrated significant reduction of NTDs [8, 10, 30]. However, the NTD risk reduction with higher blood folate concentrations is well documented, as is the enhancement of folate status with the combined consumption of folic acid supplements or fortified foods and a healthy diet containing natural folate [31–33].

Proper evidence on folate dose remains limited. For instance, the precise red blood cell folate concentration of 906 nmol/L was demonstrated to be related to a lowest risk of NTDs in the offspring. This concentration was not reached within four weeks of the currently recommended supplementation, according to Daly et al. [32]. However, Brämswig et al. were able to reach that same target level within four weeks of supplementation with a daily intake of 800 *μ*g/d folic acid. These results suggest the need for the reevaluation of the current dosage recommendation of folic acid supplementation with respect to NTD prevention [34].

#### 3.1.2. Prevention of Cardiovascular Malformations (CVMs)

Although the preventive efficacy of NTDs by folic acid-containing multivitamins (MV) or folic acid alone has been well established and demonstrated to be better than any other CA prevention, the available data also supports the essential role of folic acid for normal fetal cardiac development during early embryogenesis. The combination of the results of two Hungarian intervention trials [35, 36] (OR with 95% CI: 0.57, 0.39–0.85) showed a 43% risk reduction of cardiovascular malformations after MV supplementation.

Two more recent population-based observational studies demonstrated a significant reduction in the rates of CVM with folic acid intake [37, 38]. Furthermore, a significant reduction in the birth prevalence of severe CVMs was reported in Quebec, Canada, after folic acid fortification of grain products [39]. Another Canadian study, a systematic review and meta-analysis by Goh et al., concluded that maternal consumption of MV was associated with decreased risk for several congenital anomalies (OR 0.78, 95% CI 0.67–0.92 in case control studies and OR 0.61, 95% CI 0.40–0.92 in cohort and randomized controlled studies for cardiovascular defects) [40].

In conclusion, the available evidence concerning CVMs shows that any public health action of CA prevention with periconceptional MV or folic acid supplementation should necessarily take into consideration CVMs, with regard not only to demonstrated efficacy but also to the more elevated prevalence of CVM as compared to NTD or other defects, and thus to the superior absolute number of preventable cases of CVM per 100,000 births. This should be particularly true in countries with a low NTD prevalence, and a low NTD: CVM ratio, such as the USA.

### 3.2. Paternal Folate Deficiency

 Teratogenicity may exist at the conceptional level as well at the preconceptional level, thus affecting both maternal and paternal gametes. A recent proven example, although it has not been fully elucidated, is the effect of paternal exposure to dioxins. A statistically significant causal link has been demonstrated between dioxin exposure and spermatozoid folate deficiency leading to spina bifida [41–43].

 The chemical name for dioxin is *2,3,7,8-tetrachlorodibenzo-para-dioxin (TCDD)*. The media term “dioxins” is often used for a family of structurally and chemically related products. Dioxins are mainly unwanted byproducts of a wide range of industrial processes. In terms of dioxin release into the environment, uncontrolled waste incinerators (solid waste and hospital waste) are often the worst culprits, due to incomplete burning. Although formation is local, environmental distribution is global. Dioxins accumulate throughout the food chain, with increasing concentrations. The highest levels of these compounds are found in some soils, sediments, and food, especially dairy products, meat, fish, and poultry. Once the human body has absorbed dioxins, they persist for a long time because of their chemical stability and their ability to accumulate in fat tissue.

 After preconceptional exposure to dioxins, the risk of mutations in spermatozoids is significantly increased, leading to an increased risk of spina bifida [41–43] through mechanisms involving folate deficiency [44]. In animal models, the teratogenic and mutagenic effect of Dioxin has been extensively proven, while light had just started to be shed on the mechanism by which human paternal Dioxin exposure can lead to congenital abnormalities. The causative and statistically significant association between the Dioxin containing Agent Orange, which was used in the Vietnam War, and spina bifida, is irrefutable [41–43]. Precise biological mechanisms of exposure to Dioxin are epigenetic, based on activation of the AhR/ARNT (aryl hydrocarbon receptor/aryl hydrocarbon receptor nuclear translocator) complex of spermatogenesis, leading to folate deficiency as the cause of NTD phenotype [44, 45]. This complex is widely distributed, but is particularly abundant in the human testicle, which renders it one of the most sensitive organs to Dioxins. This explains its direct interference with human spermatogenesis and male fertility [46]. Halwachs et al. have recently demonstrated that dioxins deregulate the AhR signaling pathway [44]. This effect is mediated by a downregulation of Rfc 1 (reduced folate carrier 1) gene expression and reduced carrier protein levels. This downregulation by TCDD was shown to be time- and dose-dependent in rat livers and resulted in functional folate deficiency in male and female cells and tissues, including germ cells [44].

 In summary, dioxins are diffusely distributed in the environment and tend to accumulate along the food chain. Chronic consumption of contaminated food can lead to deregulation of genetic mechanisms implicated in folate homeostasis. Consequently, widespread folate deficiency in men and women, generally due to inadequate consumption of alimentary folates, can be increased. The final consequences are NTDs in fetuses born to mothers, but also fathers, deficient in intracellular folate concentration in germ cells. Paternal folate deficiency could be one of the factors explaining the incomplete success of recommended folate supplementation to prevent NTDs.

### 3.3. Associated Deficiencies

#### 3.3.1. Vitamin B12

 Vitamin B12 (cobalamin) is a pivotal cofactor for key enzyme reactions including the generation of methionine and tetrahydrofolate. This vitamin is found almost exclusively in foods of animal origin (meats, dairy products). Although inadequate vitamin B12 status is thought to be limited to the aging population, it has been found with a relatively high prevalence in women of reproductive age with restricted consumption of animal-based food, an increasingly popular dietary trend [16], and in pregnant women who are more likely to be deficient than nonpregnant women [47].

 Vitamin B12 deficiency may impair folate metabolism through impairment of methionine synthase enzyme. It has been associated with NTDs in a number of studies. Ray and Blom identified 17 NTD case-control studies related to B12 status, with an overall reported trend towards lower mean B12 concentration in mothers with NTD-affected pregnancies [51]. The two largest positive studies conducted after the introduction of folate fortification in the United States and Canada [52, 53] showed that the risk of NTDs was inversely proportional to the measured serum concentrations of vitamin B12 (holotranscobalamin). Furthermore, in Ireland, risk of NTDs was strongly positively correlated to low B12 status in a population not exposed to folic acid fortification or supplements [54, 55]. Kirke et al. uncovered a fivefold increase in NTD risk for women in the lower quartile of associated folate and B12 deficiencies, compared to half this rate for only folate deficiency, thus demonstrating the synergistic actions of both vitamins. More recently, Zhang et al. [56] and Molloy et al. [55] reported higher NTD risk in mothers within the lowest quartile for B12 concentrations, with an approximate three-fold increase. Both Kirke and Zhang demonstrated that B12 and folate were two independent risk factors. In 2009, the National Institutes of Health validated the results of Molloy et al., and concluded that improving B12 status in women of childbearing age would prevent NTDs. The recommended daily Vitamin B12 allowance for pregnant women is 2.6 *μ*g/day [16, 57].

 Caregivers should be aware that high levels of folic acid intake (tolerable upper intake level from fortified foods or supplements is 1000 *μ*g/d for adults), can mask vitamin B12 deficiency, resulting in permanent nerve damage, especially in elderly individuals [25].

#### 3.3.2. Other Deficiencies

 As previously mentioned, the etiology of CA is multifactorial, and most factors are still unknown. Toxic chemicals are detected everywhere, in the dietary products of the general population [58], in the inhaled air [59], and even in neonates' cord blood [60]. According to a WHO report in 2010 [61], the top three most common groups of etiologies for CA in developed countries are epigenetic: maternal diseases (diabetes and hyperthermia), pathologic maternal deficiencies (folate and iodine deficiency), and exposure to teratogens (medications, drugs including tobacco and alcohol, environmental chemicals like pesticides, and ionizing radiations).

 The unfolding of new associations between malnutrition and diseases has made it clear that women in their reproductive years have multiple complex nutritional deficiencies; this might also be true for the general population. Recent studies showed that prenatal multimicronutrient supplementation was associated with a significantly reduced risk of low birth weight when compared with iron-folic acid supplementation [62, 63]. Moreover, Chen et al. proved that periconceptional multivitamin supplementation containing folic acid—two months before conception and until completion of the second month—containing folic acid can prevent the occurrence of NTDs [64]. These maternal and paternal nutritional deficiencies are probably multicausal. They can be due to digestive malabsorption, but also to excessive cooking of fresh food that destroys most of the vitamins, and to widespread consumption of industrial food products. Future policies should be directed towards improving food quality.

## 4. Prenatal Management of NTDs

### 4.1. Prevention: Current Strategies and Their Benefits

 Epidemiological studies, both observational and interventional, have all been consistent with a 50 to 70% protective effect of adequate consumption of folates on NTDs [67]. Since strategies to modify women's dietary habits and vitamin use have achieved little progress [68] and since about half of all the pregnancies are unplanned, maternal supplementation alone cannot be an effective approach. Only maintenance of optimal nutritional status throughout the reproductive years will help ensure normal fetal development [16].

 Starting in North America, fortification of food with folic acid has made folic acid accessible to all men and women of childbearing age without necessitating behavioral change and has proven to be both efficient and more homogeneous [28]. An additional rationale for fortification is that, in contrast with food folate, bioavailability of folic acid from fortified food is 85% (it is a 100% from vitamin supplement) [25]. In 1992, the US Public Health Service (USPHS) recommended that all women in their reproductive years consume 400 *μ*g of folic acid daily for prevention of NTDs. By 1998, and following Food and Drug Administration (FDA) regulations, all standardized enriched cereal grain products sold in the United States included 140 *μ*g folic acid/100 g. Folic acid was also added to breakfast cereals, corn grits, infant formulas, medical foods, and foods for special dietary use. In 2009, the US Preventive Services Task Force published updated grade A recommendations reinforcing these guidelines [69]. By 2007, over 50 countries had implemented their own folic acid flour fortification programs, including Canada, Costa Rica, Chile, Australia, New Zealand, South Africa, and some Middle Eastern countries [70]. In the USA, the National Birth Defects Prevention Network reported a 36% decrease in the prevalence of NTDs, from 10.8 per 10,000 population during 1995-1996 to 6.9 at the end of 2006 [71]. In Europe, EUROCAT registries reported a 10% decrease, from 10.5 per 10,000 in 2004 to 9.4 in 2008 [72]. A greater decline in NTDs was predicted [73], raising the question of what additional measures should be undertaken. According to the CDC 2010 report, disparities still exist among diverse ethnicities, as well as on a global worldwide basis. As current fortification programs only prevent about 9% of total annual cases of NTDs, an international public health solution is to expand the number of countries with mandatory fortification programs that have the potential to safely diminish the fraction of folic acid-preventable NTDs [74].

 However, while the abundant literature depicts mandatory fortification programs as massive public health success stories [75], one study found that neither periconceptional supplementation nor dietary folic acid intake reduced the risk of NTDs, including spina bifida [76]. Moreover, another confirmed the protective effect of dietary folic acid alone, regardless of supplementation status, which did not appear to offer further benefit in reducing the risk of spina-bifida-affected pregnancies, even among women with very low dietary folic acid consumption [77]. It is worth mentioning here that these were case-control studies that relied on self-reported maternal questionnaires, which raises the question of reporting accuracy, in the era of global awareness of the protective effect of folic acid. Furthermore, despite folic acid fortification and maternal supplementation, the incidence of NTDs has stabilized in the United States [28] and many other countries [78].

 All this knowledge highlights the need for paired management strategy: prevention by folic acid and vitamin B12 maternal and paternal supplementations associated with a decreased exposure to the multiple risk factors and treatment such as fetal surgery.

### 4.2. Fetal Surgery

 The first prenatal myelomeningocele (MMC) repair was performed in 1994 by Bruner and Tulipan endoscopically on 4 cases. Due to premature labor and birth, this technique was considered dangerous and unsatisfactory and was abandoned [79]. In 1997, the first cases of open MMC repair by hysterotomy were realized [80, 81].

 Very recently, Adzick et al. proved, in the Management of Myelomeningocele Study (MOMS), the efficacy of fetal MMC surgery as compared to standard postnatal repair [50]. Prenatal surgery resulted in significant reduction of hindbrain herniation (Chiari II malformation), a reduction in need for shunting to relieve hydrocephalus, as well as improvements in motor function and mental development at 30 months (Table 2) [50, 82]. However, preterm delivery and obstetrical complications were increased.

## 5. Conclusion

 The etiologies of congenital abnormalities lie in epigenetics in the vast majority of cases. They are multifactorial, maternal as well as paternal, but universally associated with environmental teratogens. Future research and multicentric, large-scale trials should be directed to epigenetic profiling of congenital diseases, including neural tube defects.

 Nutritional deficiencies are multiple, interrelated, and concern both men and women. Scientific as well as political effort is mandatory in order to implement global preventive public health strategies using fortification and supplementation.

## Figures and Tables

**Table 1:** Proven and suspected risk factors for spina bifida (NP = not proven).

Risk factors: proven and certain	Relative risk
History of NTD-affected pregnancy	30
Valproic acid and carbamazepine	10–20
Pregestational maternal diabetes	2–10
Inappropriate/deficient folic acid supplementation	2–8
Paternal exposure to dioxins in Agent Orange [42]	2

Risk factors: suspected	Relative risk

Maternal vitamin B12 status	3
Maternal diarrhea	3-4
Maternal obesity	1.5–3.5
Maternal hyperthermia	2

Nonproven (NP) risk factors, with epidemiological associations	Relative risk

Gestational diabetes	NP
Pesticides	NP
Herbicides	NP
Fumonisins	NP
Water chlorination	NP
Heavy metals: lead	NP
Organic solvents	NP
Plastic byproducts (vinyl chloride, PVC)	NP
Toxic waste sights	NP
Electromagnetic field	NP
Retinoic acid (vitamin A) excess [48, 49]	NP

**Table 2:** MOM study results, January 2011 [50].

Results (%)	Prenatal surgery	Postnatal surgery	*P* value
At 12 months			
Shunt placement	40	82	<0.001
Hindbrain herniation	64	96	<0.001
At 30 months			
Psychomotor development (Bayley index mean)	64	58,3	0.03
Motor function & independent walking on examination	42	21	0.01
